# Physical development and mental health in South African perinatally HIV-positive adolescents on antiretroviral therapy and their caregivers with and without household food insecurity

**DOI:** 10.4102/sajhivmed.v22i1.1316

**Published:** 2021-12-15

**Authors:** Sarah Heany, Nicole Phillips, Landon Myer, Heather Zar, Dan Stein, Jacqueline Hoare

**Affiliations:** 1 Department of Psychiatry, Faculty of Health Sciences, University of Cape Town, Cape Town, South Africa; 2 Department of Epidemiology and Biostatistics, Faculty of Health Sciences, University of Cape Town, Cape Town, South Africa; 3 Department of Paediatrics and Child Health, Faculty of Health Sciences, University of Cape Town, Cape Town, South Africa

**Keywords:** HIV, caregiver depression, behavioural problems, hunger risk, food security, poverty

## Abstract

**Background:**

Perinatally acquired HIV-infected (PHIV+) adolescents have shown impairments in neurocognitive function and mental health problems compared with their peers. The contribution of food insecurity to such impairments has not been explored.

**Objectives:**

The aim of this report has been to explore the contribution of food insecurity to neurocognitive impairment and mental health problems in adolescents with perinatally-acquired HIV infection.

**Method:**

A total of 248 PHIV+ adolescents and healthy controls aged between 9 and 12 years completed a neuropsychological battery, the Childhood Behaviour Checklist (CBCL) and the Becks Youth Inventory. Head circumference, body mass index (BMI), height for age (HAZ), Tanner pubertal staging, albumin, haemoglobin, CD4 and viral loads were also measured. Participants’ caregivers were interviewed about their mental health and household food security. T-tests were used to assess for differences in food secure and food insecure households.

**Results:**

Caregivers of PHIV+ adolescents reported higher levels of depressive symptoms and household food insecurity. Increased food insecurity was associated with more behavioural problems in adolescents, as well as lower haemoglobin and albumin levels, faster processing speed and increased Tanner staging in boys. Body mass index and HAZ were not affected by food insecurity.

**Conclusion:**

These findings suggest that household food insecurity is associated with some altered behavioural, physical and physiological outcomes, which could complicate and compound the existing difficulties in PHIV+ households.

## Introduction

Adolescents with perinatally acquired HIV infection (PHIV+) have worse outcomes in general cognitive development and mental health compared with age-matched HIV-negative peers,^1,2,3,4,5^ with the socioeconomic status as a potential cofounder.^6^ These include a poorer function in cognitive domains, such as working memory, executive function and processing speed,^2^ and a higher prevalence of attention deficit hyperactivity disorder (ADHD) symptoms, depressive symptoms, anger, disruptive behaviour, self-concept or functional competence.^4^ Both physiological and socio-environmental conditions have been implicated in these associations, in youth and adolescents, specifically with increased stress or mental ill health in their caregivers.^7^ A South African study^8^ found that depression amongst caregivers was strongly associated with problematic adolescent behaviour, more so than the HIV status.

Cognitive and behavioural problems in youth are also aggravated in situations of poverty.^9^ Food insecurity specifically has been associated with increased cognitive and behavioural problems in children and adolescents. Both externalising and internalising subscales of the Childhood Behaviour Checklist (CBCL),^10,11^ as well as behaviour reports from teachers,^12^ have been associated with food insecurity. In addition, food insecurity has been associated with lower antiretroviral therapy (ART) adherence in rural Ugandan HIV-affected households.^13^ South African PHIV+ adolescents also often experience high rates of orphanhood and living with extended family, often in unstable living environments.^14^

Studies examining problematic or disruptive behaviour in PHIV+ adolescents are sparse.^15^ In addition, the impact of food insecurity on measures of cognition, affect, and behaviour may play a moderating role in the developing mental health of adolescents, but such findings have not been reported in the existing literature. In this study, we investigated the effects of household food insecurity on mental health conditions of adolescents and caregivers.

## Methods

### Participants and setting

This is a cross-sectional analysis of enrolment data from a neuropsychiatric sub-study of the Cape Town Adolescent Antiretroviral Cohort (CTAAC), a study of PHIV+ adolescents on ART in a large city in South Africa. The neuropsychiatric sub-study recruited PHIV+ adolescents from ART services across the municipality. These adolescents were eligible to participate if they were aged 9–12 years, were on ART for > 6 months and knew their HIV status. HIV-negative healthy controls (HCs) were frequency matched with PHIV+ participants on age, sex and ethnicity. All adolescents screened for the HC cohort underwent rapid HIV testing to confirm their negative status. The exclusion criteria for both groups were as follows: (1) an uncontrolled medical condition, such as diabetes mellitus, epilepsy and tuberculosis (TB) requiring hospital admission; (2) an identified central nervous system (CNS) condition, including meningitis (TB or bacterial), cerebrovascular accident, lymphoma, history of head injury with loss of consciousness greater than 5 min or any radiological evidence of skull fracture, history of perinatal complications, including hypoxic ischaemic encephalopathy or neonatal jaundice requiring exchange transfusion, or neurodevelopmental disorder not attributed to HIV. The primary caregiver of each participant provided written informed consent prior to participation, and each participant provided his or her assent for participation. All data were obtained from 204 PHIV+ and 44 HC participants between 2013 and 2015.

### Measures

Adolescents had a clinical examination, including Tanner staging and anthropometry. A blood sample for measuring albumin, CD4 and viral load (VL) was taken. Weight was measured in kilograms on a Scales 2000 calibrated digital scale to the nearest 0.1 kg. The standing height was measured in centimetres using a stadiometer with a moveable headboard to the nearest 0.1 cm.^16^ Body mass index (BMI) was calculated from the measured height and weight as weight/height^2 (kg/m^2). In order to assess for stunting, height for age Z scores (HAZ) were calculated separately for girls and boys using the 2007 WHO growth reference for school-aged children and adolescents (available at http://www.who.int/growthref/tools/en/). Weight for age Z scores are not recommended for children over the age of 10 years.

Adolescents completed the following measures: Beck Youth Inventories^17^ was used to assess depression, anxiety, anger, disruptive behaviour and self-concept. Each participant was assessed using a battery of standardised neuropsychological tests, including measures of processing speed and working memory, as used in paediatric assessment and research in South Africa.^2^ Antiretroviral therapy adherence was recorded using a self-report questionnaire. Tests were administered in the adolescents’ home language.

Caregivers completed the following measures regarding the adolescents’ behaviour: The CBCL^18^ to assess their adolescents’ behavioural and emotional problems and psychopathology using four subscales: total problems, total competence, internalising behaviour and externalising behaviour. Conners Parent Rating Scale^19^ was used to assess ADHD symptoms. Adolescent ART adherence was also reported on by the caregivers.

Caregivers completed the following measures regarding their own experiences: Caregiver depression was measured using the Center for Epidemiological Studies-Depression (CES-D).^20^ The Community Childhood Identification Project Hunger Index (CCHIP) food security scale, as used in the South African National Health and Nutrition Examination 2012,^21^ was used to assess household food security. Scores were obtained via an eight-point self-reported questionnaire as completed by the legal guardian of the adolescent (see Appendix 1). A score of 0 indicates food secure and a score of > 1 indicates food insecure.

Center for Epidemiological Studies-Depression, CBCL, household poverty, cognitive performance, and Tanner staging (indicating pubertal development), were assessed at baseline. Food security data were collected at a 6-month follow-up. Albumin and haemoglobin (Hb) levels were recorded 12 months after the baseline.

### Statistics

Differences in mental health, cognition and behaviour in the adolescents, as well as depression of the caregiver, were assessed between the food security groups (food secure and food insecure). These tests were carried out in the combined sample (PHIV+ and HC together) and in the PHIV+ group alone. The tests were not carried out in the HC group alone as there were not enough HC participants in the food insecure category to facilitate comparative tests. Mean measures of albumin, Hb and physical development were assessed between the food secure and food insecure groups. Differences in the above measures were also assessed between the PHIV+ and HC groups. The ART adherence scores were tested for differences based on food security levels. The different measures of disruptive behaviour (Becks Disruptive Behaviour [BDB] and CBCL subscales) were tested for correlations. Statistical tests were conducted using Statistical Package for the Social Sciences (SPSS) for Windows, version 26 (IBM Corp., Armonk, New York, United States). For all tests of interval data, *t*-test was used. For tests of categorical data (Tanner staging, home language and repeated grades), chi-square tests were used. Levene’s test for equality of variances was assessed for each *t*-test to determine whether a Mann–Whitney test was required. In post hoc and sensitivity testing, some behavioural and affective measures were assessed for correlations using Pearson’s correlation coefficients.

### Ethical considerations

This study was approved by the University of Cape Town’s Faculty of Health Sciences Human Research Ethics Committee (HREC REF 051/2013) and is in accordance with the Declaration of Helsinki. All data were obtained from 204 PHIV+ and 44 HC CTAAC participants between 2013 and 2015.

## Results

### Overall results in the full sample

The mean annual income was just under $350.00, on average, for both PHIV+ and HC households, classified as extreme poverty (< $1.90 per day) by the World Bank.^22^ The median age of the sample (including PHIV+ and HC) was 10.8 years. Tanner staging was higher in girls than boys. A total of 225 adolescent caregivers provided food security scores, with 50 indicating food insecurity. The average age of ART onset in the PHIV+ group was 3.4 years, with a standard deviation of 2.53. In the PHIV+ group, the median CD4 count was 903 cells/mm^3^, and the mean was 957 cells/mm^3^. About 17% of the PHIV+ group had detectable VL levels of over 50 copies/mL. The findings as well as differences between the PHIV and HC groups have been listed (Table 1).

**TABLE 1:** Baseline demographic and clinical characteristics of the cohort, comparing perinatally acquired HIV infection and healthy controls groups.

Variable	PHIV+ (*N* = 203)			HC (*N* = 43)			*P*
	Mean	s.d.	%	Mean	s.d.	%	
**Age (years)**	10.79	0.9	-	10.72	1.0	-	0.65
**Gender**	-	-	-	-	-	-	0.73
Male	94	-	-	19	-	-	-
Female	104	-	-	24	-	-	-
Height, (cm)	133.9	7.7	-	138.5	8.8	-	< 0.01*
HAZ scores	−1.24	1.0	-	−0.35	1.0	-	< 0.01*
Weight, (kg)	30.8	7.3	-	36.7	11.4	-	< 0.01*
BMI	17.1	3.0	-	18.8	3.8	-	< 0.01*
Head circumference, (cm)	52.9	2.1	-	53.6	3.1	-	0.09
**Tanner stage (boys), % at each stage**	-	-	-	-	-	-	0.54
State 1	-		90	-	-	84	-
Stage 2	-	-	8	-	-	16	-
Stage 3	-	-	1	-	-	-	-
**Tanner stage (girls), % at each stage**	-	-	-	-	-	-	0.01*
State 1	-	-	52	-	-	21	-
Stage 2	-	-	36	-	-	63	-
Stage 3	-	-	9	-	-	8	-
Stage 4	-	-	2	-	-	8	-
Haemoglobin (Hb), g/dL	12.5	1.1	-	12.8	1.1	-	0.18
Albumin, g/L	42.9	3.5	-	45.1	2.9	-	< 0.01*
Viral load, copies/mL: % that have values > 50	-	-	17	n/a	-	-	n/a
CD4 count, cells/mm^3^	957	484	-	n/a	-	-	n/a
Home language: % isiXhosa speakers	-	-	90	-	-	95	0.08
Household income bracket†	2.9	0.7	-	2.7	0.5	-	0.21
Food insecurity	1.1	2.3	-	0.2	1.3	-	0.02*
**Current school grade**	4.2	1.4	-	4.4	1.1	-	0.24
**Repeated grades: % that have repeated**	-	-	59	-	-	42	0.003*
**Caregiver depression (CES-D)**	16.0	12.4	-	11.3	10.8	-	0.02*

Note: *P-*values are provided from *t*-tests carried out for interval data and chi-square tests for nominal data. Values are reported as mean (s.d.) unless indicated otherwise.

PHIV+, perinatally acquired HIV infection; HC, healthy controls; BMI, body mass index; CES-D, Center for Epidemiological Studies-Depression; HAZ, height for age.

* , Statistical significance with a threshold of *P* < 0.05.

† , Household annual income brackets: 1: $0.00; 2: $1.00–$350.00; 3: $351.00–$1500.00; 4: > $1500.00.

### Differences between perinatally acquired HIV infection and healthy controls groups

Levels of poverty were well-matched between the PHIV+ and HC groups. Despite matched poverty levels, 26% of PHIV+ households experience food insecurity, whilst only 5% of HC households reported food insecurity. The number of repeated grades differed between the groups, with 59% of the PHIV+ group and 42% of the HC group having repeated a grade. Albumin levels were lower in the PHIV+ group. Body weight, height, HAZ, BMI and female Tanner scores were all lower in the PHIV+ group. The median HAZ score in the PHIV+ group was –2 and in the HC group was –1.9. Caregiver depression and food insecurity were higher in the PHIV+ group. In the PHIV+ group, 203 caregivers provided CES-D scores, 94 of whom are considered at risk for depression (a score of ≥ 16).^23^ In the HC group, 44 parents completed the CES-D, 13 of whom had a score of ≥ 16.

### Affect and cognitive scores by food insecurity (Table 2)

In the combined sample (PHIV+ and HC group), Hb and albumin levels were lower in the food insecure group. Problem behaviour, as measured by CBCL Total Problems (CBCL-TP), was higher in the food insecure group (*t* = 2.05(220), *P* = 0.042). Cognitive domains and BMI did not differ according to food insecurity in the combined sample. The BDB measure of disruptive behaviour almost differed (*P* = 0.078), whilst depression, anxiety and anger did not differ. A test correlating BDB scores with CBCL scores showed that BDB was correlated with CBCL TP (*r* = 0.23, *P* < 0.001), but not with the other CBCL subscales (externalising behaviour, internalising behaviour and total competence). Tanner stage in boys was slightly higher in the food insecure group, although boys’ ages were very closely matched (10.7 years in the food secure group and 10.8 years in the food insecure group). When the subscales of the CBCL were compared between the food secure and food insecure groups, only CBCL-TP was significantly different.

**TABLE 2:** Baseline demographic and clinical characteristics of the cohort, comparing food insecure and food secure.

Variable	Combined group (PHIV+ and HC)						*p*	PHIV+ only						*p*
	Food insecure (*N* = 50)			Food secure (*N* = 174)				PHIV+ Food insecure (*N* = 48)			PHIV+ Food secure (*N* = 135)			
	Mean	s.d.	%	Mean	s.d.	%		Mean	s.d.	%	Mean	s.d.	%	
Age (years)	10.8	0.9	-	10.8	0.9	-	0.54	10.8	0.9	-	10.8	0.8	-	0.95
**Gender**	-	-	-	-	-	-	0.94	-	-	-	-	-	-	0.84
Male	24	-	-	83	-	-	-	23	-	-	67	-	-	-
Female	26	-	-	92	-	-	-	25	-	-	68	-	-	-
Height (cm)	134.8	7.9	-	134.5	7.9	-	0.84	134.7	8.1	-	133.4	7.3	-	0.32
HAZ scores	−1.22	1.0	-	−1.08	1.1	-	0.40	−1.24	1.0	-	−1.28	1.0	-	0.84
Weight (kg)	31.0	5.5	-	32.1	9.1	-	0.44	30.8	5.5	-	30.8	7.8	-	0.95
BMI	16.9	2.0	-	17.5	3.6	-	0.30	16.9	1.9	-	17.2	3.5	-	0.51
Head circumference	52.8	2.3	-	53.1	2.4	-	0.40	52.8	2.4	-	53.0	2.1	-	0.52
**Tanner stage (boys) % at each stage**	-	-		-	-	-	0.04*	-	-	-	-	-	-	0.15
Stage 1	-	-	74	-	-	93	-	-	-	77	-	-	94	-
Stage 2	-	-	26	-	-	6	-	-	-	23	-	-	5	-
Stage 3	-	-		-	-	1	-	-	-	-	-	-	1	-
**Tanner stage (girls) % at each stage**	-	-		-	-	-	0.07	-	-	-	-	-	-	0.26
Stage 1	-	-	62	-	-	40	-	-	-	60	-	-	48	-
Stage 2	-	-	30	-	-	47	-	-	-	32	-	-	39	-
Stage 3	-	-	8	-	-	10	-	-	-	8	-	-	10	-
Stage 4	-	-		-	-	3	-	-	-	-	-	-	3	-
HB g/dL	12.3	1.3	-	12.7	1.1	-	0.05*	12.4	1.3	-	12.6	1.1	-	0.14
Albumin g/L	42.4	3.7	-	43.7	3.3	-	0.02*	42.5	3.7	-	43.2	3.3	-	0.22
Viral load copies/mL: % > 50 PHIV+ only	-	-	17	-	-	15%	0.32	-	-	17	-	-	15	0.32
CD4 count cells/mm^3^ PHIV+ only	960	445	-	931	446	-	0.70	960	445	-	931	446	-	0.70
ART adherence (self-reported; PHIV+ only)	14.54	2.3	-	14.65	2.0	-	0.76	14.54	2.3	-	14.65	2.0	-	0.76
ART adherence (parent reported; PHIV+ only)	14.29	2.0	-	14.46	2.0	-	0.64	14.29	2.0	-	14.46	2.0	-	0.64
Household income bracket†	2.8	0.6	-	2.9	0.7	-	0.41	2.8	0.6	-	2.9	0.7	-	0.21
Food insecurity	4.1	2.8	-	0	0	-	< 0.01*	4.1	2.8	-	0	0	-	< 0.01*
Current school grade	3.2	1.2	-	3.2	1.2	-	0.90	3.2	1.2	-	3.2	1.1	-	0.78
**Repeated grades**	-	-	-	-	-	-	0.57	28/20	-	-	-	-	-	0.84
Yes	30	-	-	97	-	-	-	-	-	-	81	-	-	-
No	20	-	-	78	-	-	-	-	-	-	54	-	-	-
Caregiver depression (CES-D)	17.1	11.7	-	14.5	12.2	-	0.18	17.6	11.6	-	15.5	12.4	-	0.32
Child Behavior Checklist – Total problems (CBCL-TB)	58.0	11.4	-	53.9	11.0	-	0.02*	58.0	11.6	-	54.5	11.5	-	0.07
Child behavior checklist – Total competence	38.7	8.0	-	38.1	8.0	-	0.62	38.6	7.8	-	37.3	7.9	-	0.33
Child behavior checklist – Internalising problems	58.6	12.1	-	56.7	10.7	-	0.29	58.8	12.3	-	57.0	11.1	-	0.36
Child behavior checklist – Externalising problems	55.2	10.9	-	52.3	10.8	-	0.10	55.0	11.0	-	52.9	11.0	-	0.26
ADHD (Conners)	17.0	14.0	-	14.4	14.6	-	0.79	16.8	13.9	-	15.6	15.2	-	0.39
Beck’s self-Concept	45.9	10.1	-	46.6	8.6	-	0.65	45.3	9.8	-	46	8.9	-	0.67
Beck’s anxiety	54.7	13.4	-	53.1	13.1	-	0.46	55.0	13.6	-	52.9	13.7	-	0.36
Beck’s depression	48.1	11.9	-	45.4	10.3	-	0.12	48.6	11.9	-	46.4	10.6	-	0.22
Beck’s anger	46.8	13.7	-	43.8	11.2	-	0.11	47.4	13.7	-	44.0	11.6	-	0.11
Beck’s disruptive behaviour (BDB)	46.1	8.9	-	43.5	9.4	-	0.08	46.5	8.9	-	44.2	9.6	-	0.16
Processing speed (Z score)	−0.42	0.7	-	−0.48	0.7	-	0.60	−0.40	0.7		−0.63	0.7	-	0.04*

Note: *P*-values are provided from *t*-tests carried out in the case of interval data, and chi-square tests in the case of nominal data. Values are reported as mean (s.d.) unless indicated otherwise.

PHIV+, perinatally acquired HIV infection; HC, healthy controls; BMI, body mass index; CES-D, Center for Epidemiological Studies-Depression; HAZ, height for age; Hb, haemoglobin; ART, antiretroviral therapy; ADHD, attention deficit hyperactivity disorder; s.d., standard deviation.

* , Statistical significance with a threshold of *P* < 0.05.

† , Household annual income brackets: 1: $0.00; 2: $1.00–$350.00; 3: $351.00–$1500.00; 4: > $1500.00.

### Affect and cognitive scores by food insecurity amongst perinatally acquired HIV-infected adolescents

When the PHIV+ group was investigated on its own, problem behaviour as measured by CBCL-TP was no longer significantly different between the food security group (*t* = 1.82(180), *P* = 0.07). It should be mentioned that this change in *P*-value from 0.04 to 0.07 (along with a change in *t*-score from 2.05 to 1.82) may be because of the smaller *n* in this test of this subsample. Amongst the cognitive domains, only processing speed differed amongst the food security groups, with food insecurity being associated with faster processing speed. We noted that processing speed was not different between the food secure and food insecure groups when the PHIV+ and HC were combined (*t* = –0.53, *P* = 0.60), only within the PHIV+ sample, as shown in Table 2. There was no difference in reported ART adherence between the food secure and food insecure groups.

## Discussion

This study of the CTAAC adolescent cohort found that food insecurity was associated with problem behaviour, as measured by CBCL-TP. In addition, processing speed was faster in PHIV+ adolescents who experience household food insecurity. We also found that food insecurity was *not* associated with working memory, CBCL Total Competence or Conners ADHD scale. Becks inventory scores of depression, anxiety, anger and disruptive behaviour were also not significantly associated with food security, although the data trended towards the food insecure groups having higher scores in each of these inventory domains. In the whole sample (PHIV+ and HC combined), lower Hb and albumin levels were associated with food insecurity. Perinatally acquired HIV infection households were more likely to have food insecurity and caregiver depression. Lower height, weight, BMI and albumin levels were also related to PHIV status.

Problem behaviour differed according to level of household food insecurity. Becks Disruptive Behaviour and CBCL-TP also correlated strongly. It is, therefore, possible that both being PHIV+ and having food insecurity in the home may compound adolescent problem behaviour. A longitudinal study conducted in the United States found that whilst there are higher rates of problem behaviour in HIV+ children, demographic factors such as level of parental education may be a stronger cause of problem behaviour than HIV diagnosis.^24^ A study on a South African cohort of PHIV+ children and adolescents also found that problem behaviour was not directly linked to HIV status, but instead was more associated with caregiver depression.^8^ This study adds to these findings by including household food insecurity and shows that it may have a modulating effect on problem behaviour in adolescents. Overall, the literature points to a complex interaction of social and demographic factors that influence problem behaviour in PHIV+ and healthy adolescents. Interestingly, a study from Atlanta, Georgia, on HIV+ children and adolescents (97% perinatally infected) and controls showed that whilst psychological maladjustment was high in the HIV+ group, it was even higher in the control group, specifically with regard to internalising behaviour.^25^ Problem behaviour, particularly problematic conduct, has previously been associated with decreased ART adherence in PHIV+ children and adolescents in the United States.^26^ Food insecurity has also been associated with lower ART adherence in rural Ugandan HIV+ households.^13^ Whilst this may have severe implications for the ongoing health and HIV+ population, our PHIV+ sample did not show any differences in reported adherence in relation to food insecurity.

In our cohort, height, weight, BMI, HAZ and Tanner staging in girls were all lower in the PHIV+ group, indicating stunted growth; however, these growth biomarkers were not related to food insecurity. A study in Tanzanian adolescents has previously associated food insecurity with undernutrition and lower BMI, although mental health was not measured.^27^ Alternately, in the North American populations, food insecurity has been associated with an increased risk of obesity and metabolic syndrome in both adults and adolescents.^28,29,30,31^ This inconsistency in published results may be because of different lifestyle norms and factors of poverty in each studied population. A review focussing on Western countries found that food insecurity had deleterious consequences of emotional, cognitive and behavioural outputs in children and adolescents.^32^ A UK-based study found that cognitive differences in food insecurity groups could be explained by poverty levels, and behavioural development could be explained by environmental and parental treatment in the home; emotional problems were, however, ascribed to food insecurity alone.^33^ This study tries to piece these different factors together; however, more longitudinal data are required.

There are some limitations to this study. One is the cross-sectional design; however, follow-up analyses with this cohort are underway. The food security data, household income, mental health, and problem behaviour measures in this study were all self-reported by parents or legal guardians, which leaves some room for error, given that answers rely on recall. Responses to measures, such as the food security questionnaire, are vulnerable to biased reporting from participants. This bias can depend on the participants’ comfort and familiarity with the data collection team, and may be influenced by inhibition or alternately a tendency towards expected responses when facing many questions about experienced difficulties. Another limitation here is the small size of the HC group; however, the group was well matched. Different measures of food insecurity are used in research studies across the world because of different living conditions and norms; however, this currently seems necessary, given differing socio-cultural contexts. In order to clarify the associations between problem behaviour, HIV status, poverty indicators, nutrition, development and mental states, the effects of specific family and household dynamics of affected children and adolescents should be studied further, especially using objective food security measures and with research designs that facilitate understanding of causal pathways.

In conclusion, PHIV+ adolescents suffer delays in physical development, including BMI, HAZ, pubertal development and lower circulating albumin levels. Household food insecurity further affects albumin and Hb levels and increases the likelihood of behavioural problems in PHIV+ adolescents.

## Appendix

**APPENDIX 1:** The community childhood identification project hunger index food security scale.

Please answer ‘Yes’ or ‘No’ to each of the following questions:			
1.	Does your household ever run out of money to buy food?	■ Yes	■ No
2.	Do you ever rely on a limited number of foods to feed your children because you are running out of money to buy food for a meal?	■ Yes	■ No
3.	Do you ever cut the size of meals or skip any because there is not enough food in the house?	■ Yes	■ No
4.	Do you ever eat less than you should because there is not enough money for food?	■ Yes	■ No
5.	Do your children ever eat less than you feel they should because there is not enough money for food?	■ Yes	■ No
6.	Do your children ever say they are hungry because there is not enough food in the house?	■ Yes	■ No
7.	Do you ever cut the size of your children’s meals or do they ever skip meals because there is not enough money to buy food?	■ Yes	■ No
8.	Do any of your children ever go to bed hungry because there is not enough money to buy food?	■ Yes	■ No
